# Ectopic Lipid Accumulation Correlates with Cellular Stress in Rabbit Blastocysts from Diabetic Mothers

**DOI:** 10.3390/ijms241411776

**Published:** 2023-07-21

**Authors:** Maria Schindler, Sophia Mareike Geisler, Tom Seeling, Anne Navarrete Santos

**Affiliations:** Institute of Anatomy and Cell Biology, Faculty of Medicine, Martin Luther University, 06108 Halle, Germanyanne.navarrete-santos@uk-halle.de (A.N.S.)

**Keywords:** embryoblast, trophoblast, lipid metabolism, fatty-acid uptake, preimplantation embryo, oxidative stress

## Abstract

Maternal diabetes mellitus in early pregnancy leads to hyperlipidemia in reproductive tract organs and an altered embryonic environment. To investigate the consequences on embryonic metabolism, the effect of high environmental-lipid levels was studied in rabbit blastocysts cultured with a lipid mixture in vitro and in blastocysts from diabetic, hyperlipidemic rabbits in vivo. The gene and protein expression of marker molecules involved in lipid metabolism and stress response were analyzed. In diabetic rabbits, the expression of embryoblast genes encoding carnitine palmityl transferase 1 and peroxisome proliferator-activated receptors α and γ increased, whereas trophoblast genes encoding for proteins associated with fatty acid synthesis and β-oxidation decreased. Markers for endoplasmic (activating transcription factor 4) and oxidative stress (nuclear factor erythroid 2-related factor 2) were increased in embryoblasts, while markers for cellular redox status (superoxide dismutase 2) and stress (heat shock protein 70) were increased in trophoblasts from diabetic rabbits. The observed regulation pattern in vivo was consistent with an adaptation response to the hyperlipidemic environment, suggesting that maternal lipids have an impact on the intracellular metabolism of the preimplantation embryo in diabetic pregnancy and that embryoblasts are particularly vulnerable to metabolic stress.

## 1. Introduction

The preimplantation stage of embryo development is a period of well-orchestrated molecular events, starting with fertilization of the oocyte and ending with the implantation of the embryo in the uterus [1]. During this period of time, the embryo goes from a relatively inactive metabolic tissue at ovulation to a rapidly metabolizing tissue at implantation [2]. Understanding the embryo metabolism during these first steps of development is critical for deciphering long-lasting effects. For example, maternal metabolic diseases like diabetes mellitus can impair preimplantation development and reprogram offspring metabolism, affecting postnatal growth trajectories [3]. Diabetes mellitus, which is characterized by hyperglycemia as well as hyperlipidemia [4,5,6], alters normal cellular metabolism and signaling. Animal studies have shown that maternal diabetes mellitus can contribute to offspring metabolic programming at very early stages of development through molecular and structural changes to preimplantation embryos [7,8]. In this context, embryonic lipid metabolism is increasingly attracting scientific research [9].

Cellular lipid metabolism is tightly controlled by transcription factors, especially peroxisome proliferator-activated receptors (PPARs). Fatty acids and lipid metabolites can serve as endogenous PPAR ligands to exert an adaptive metabolic response to changes in metabolism [10]. PPARα and PPARγ act as key transcription factors of lipid metabolism and regulate a wide range of genes, including the key enzyme for β-oxidation, carnitine O-palmitoyltransferase 1 (CPT1), as well as fatty acid binding proteins (FABPs) and fatty acid transport proteins (FATPs) [11,12]. In addition, PPARs exert indirect effects on intracellular lipid metabolism via the transcription factor sterol regulatory element-binding protein 1c (SREBP1c), and thereby regulate the key enzymes for de novo lipogenesis: acetyl-CoA carboxylase (ACC), fatty acid synthase (FASN), and stearoyl-CoA desaturase 1 (SCD1) [13,14,15]. The effects of PPAR signaling are directly related to their interaction with co-regulators. For example, PPARγ coactivator 1α (PGC1α), which interacts with both PPARα and PPARγ, plays a pivotal role in the regulation of cellular energy metabolism [16].

In addition to metabolic changes, PPAR signaling also impacts redox status by regulating mitochondrial biogenesis and anti-oxidant defense [17]. PPARγ and PPARα are involved in the regulation of mitochondrial superoxide dismutase 2 (Sod2), which has a crucial role in regulating cellular redox status [18,19,20]. Numerous studies have shown direct and indirect interactions between PPARγ and nuclear factor erythroid 2-related factor 2 (Nrf2, also named NFE2L2) in the regulation of oxidative stress defense and mitochondrial biogenesis [21]. Together with PGC1α, PPARγ activates Nrf2 and the mitochondrial transcription factor A. The activation of both factors leads to the synthesis of mitochondrial DNA and proteins, which eventually results in the biogenesis of new mitochondria [22]. Heat shock protein 70 (Hsp70) also serves as a cytoplasmic antioxidant by shielding sensitive sites of target proteins [23]. Cell lines overexpressing Hsp70 are protected against oxidative stress and apoptotic stimuli [24,25]. Hyperglycemia and diabetes mellitus are associated with higher Hsp70 levels in human serum [26]. Endoplasmic reticulum (ER) stress has been observed in embryos from diabetic pregnancies [27,28,29]. One important marker of ER stress is cyclic AMP-dependent transcription factor ATF-4 (ATF4) [30]. In a previous study, ATF4 expression was highly increased in embryoblast (EB) cells, the inner cell mass of blastocysts, from diabetic rabbits [31].

The metabolic capacities of the EB and trophoblast (TB; trophectoderm tissue) are different. In mouse blastocysts, the inner cell mass is metabolically “quieter” than the trophectoderm [32]. Cells of the trophectoderm are responsible for creating the environment within the blastocoel, consume more oxygen, and appear to have more mitochondria [33]. In rabbits, the EB and TB cells respond differently to metabolic challenges imposed by maternal diabetes mellitus [34,35]. For example, EB cells tend to accumulate intracellular lipid droplets and predominantly express FABP4 and adipophilin genes [35]. In addition, the metabolic profiles of the EB and TB cells are differentially affected by maternal diabetes mellitus [36]. While saturated fatty acids (palmitic and stearic acid) were elevated in the EB cells of diabetic rabbits, polyunsaturated fatty acids, such as docosahexaenoic acid, were decreased [36]. In contrast, lower levels of palmitic and stearic acid and higher levels of oleic acid were observed in the TB cells [36].

To determine the potential consequences of cellular stress adaptation, we investigated embryonic lipid metabolism in EB and TB cells separately in rabbit blastocysts from diabetic pregnancies. Diabetes mellitus is associated with maternal and fetal dyslipidemia, which manifests as high plasma triglyceride concentrations, elevated concentrations of nonesterified fatty acids, increased concentrations of low-density lipoprotein cholesterol, and decreased levels of high-density lipoprotein cholesterol [37,38]. To show that the observed changes are due to the maternal hyperlipidemia [35], we used in vitro experiments that analyzed the effect of the hyperlipidemic environment.

## 2. Results

### 2.1. Expression of Key Lipogenic Markers in Blastocysts

**Figure 1:**
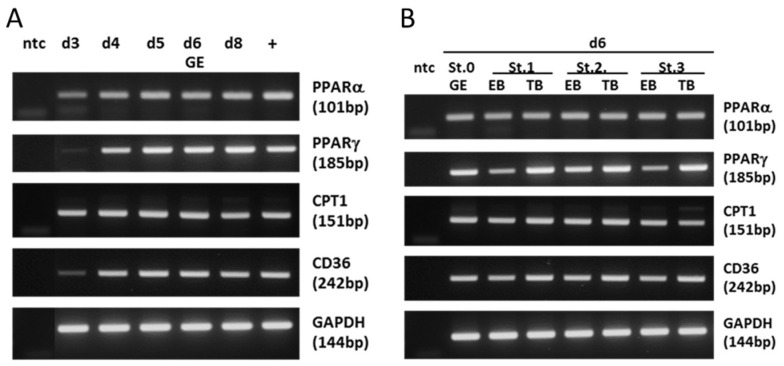
mRNA expression pattern of lipogenic marker genes. (**A**) RT-PCR was performed with specific primers for PPARα, PPARγ, CPT1, and CD36 genes on cDNA from early rabbit embryos. In (**A**): morulae at day (d) 3, blastocysts at d4, d5, d6, and d8 post coitum. In (**B**): non-gastrulated 6-day-old blastocysts at stage 0 (St. 0) and 6-day-old blastocysts at gastrulation stages 1, 2, and 3 (St. 1–3), In gastrulating blastocysts, RT-PCR was performed separately in embryoblasts (EBs) and trophectoderms (TBs). A sample without cDNA was used as negative control (ntc) in each experiment. A liver cDNA probe was used as a positive control (+). cDNA integrity was verified with RT-PCR on the GAPDH gene in all probes. (bp: base pairs).

The gene expression of key signaling molecules regulating intracellular lipid metabolism was analyzed, including PPARα (gene name: PPARA), PPARγ (PPARG), CPT1, and CD36 in rabbit morulae (day 3 post coitum (p.c.)) and peri-implanting blastocysts, using reverse transcription-polymerase chain reaction (RT-PCR) (Figure 1).

Transcripts of PPARα, PPARγ, CPT1, and CD36 were detected in all analyzed developmental stages. At the morula stage on day 3 p.c., PPARγ and CD36 had relatively lower band intensities compared with the blastocyst stage, indicating that the expression of these genes increased during development. Expression levels of PPARγ were different in TBs, with a higher level of PPARγ in TBs.

### 2.2. PPAR Expression in Blastocysts from Diabetic Rabbits In Vivo

**Figure 2:**
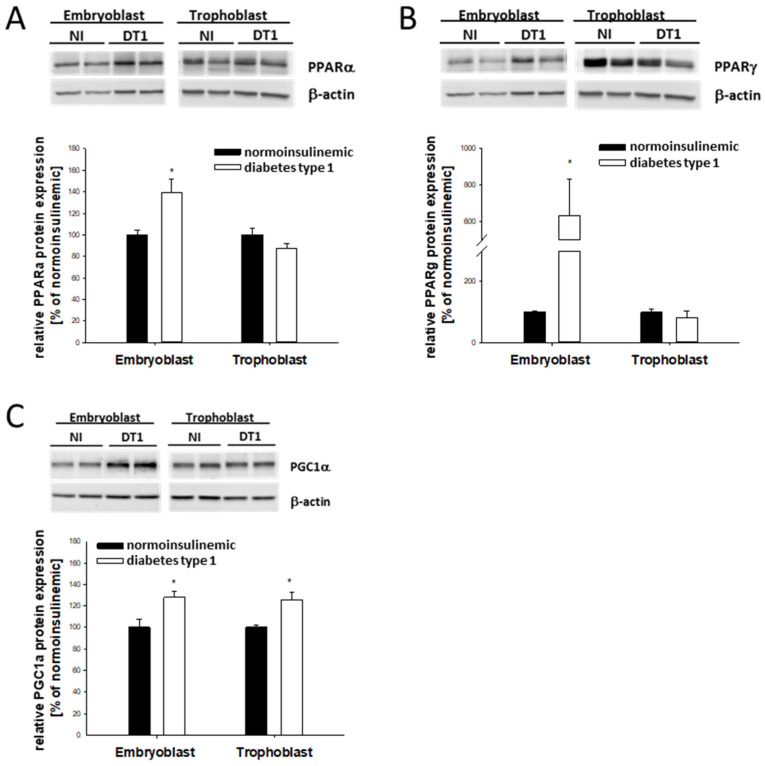
Relative protein abundance of PPARγ, PPARα, and PGC1α in blastocysts from diabetic rabbits. Protein abundance of PPARα (**A**), PPARγ (**B**), and PGC1α (**C**) was quantified in 6-day-old blastocysts from diabetic [DT1, white bars] and normoinsulinemic [NI, black bars] rabbits. For Western blot analysis, samples from at least three independent experiments with 8 to 10 blastocysts per sample were used (N ≥ 3; *n* = 8–10). Representative Western blots are shown with two samples per treatment group and cell lineages. Band intensities were measured using densitometry and normalized to levels of β-actin in the same membrane. In the graphs, protein amounts are relative to normoinsulinemic controls (100%). Values are expressed as mean ± SEM in % of non-diabetic controls. (*n* ≥ 8; * *p* < 0.05).

Maternal diabetes type 1 alters the expression of PPARα and PPARγ in gastrulating blastocysts, depending on the cellular lineage. In EB cells, PPARα and PPARγ protein levels were significantly increased (Figure 2A,B), whereas levels remained constant in the TB cells.

PPARγ protein level correlated with the mRNA abundance (Table 1), while the transcription of PPARα was not significantly altered.

PPARα protein expression results were confirmed using whole-mount immunofluorescence (Supplemental Data, Figure S1). PPARα signal intensity was stronger in EB than TB cells in both normoinsulinemic and diabetic animals.

In addition, we analyzed the expression of PGC1α, the transcriptional coactivator of PPARα and PPARγ, in blastocysts from diabetic rabbits. PGC1α protein abundance was increased in both EB and TB cells (Figure 2C).

### 2.3. Lipogenic Marker Expression in Blastocysts from Diabetic Rabbits In Vivo

**Figure 3:**
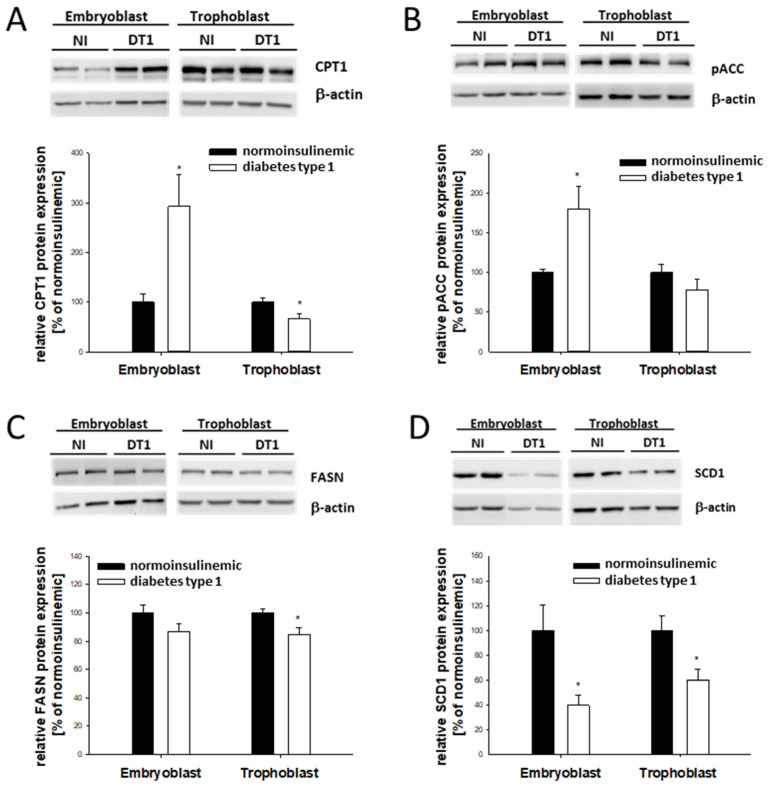
Relative protein abundance of CPT1, phosphorylated ACC, FASN, and SCD1 in blastocysts from diabetic rabbits. Protein abundance of CPT1 (**A**), phosphorylated ACC (**B**), FASN (**C**), and SCD1 (**D**) was quantified in 6-day-old blastocysts from diabetic [DT1, white bars] and normoinsulinemic [NI, black bares] rabbits. Representative Western blots are shown with two experimentally independent samples. For quantification, samples from at least three independent experiments with 8 to 10 blastocysts per sample were used (N ≥ 3; *n* = 8–10). Relative amounts are shown in bar diagrams after normalization relative to β-actin levels. Values are expressed as mean ± SEM in % of non-diabetic controls. (*n* ≥ 8; * *p* < 0.05).

While CPT1 was increased 3-fold in EB cells, expression was significantly decreased in TB cells (Figure 3A), indicating that the overall increase resulted from changes in the EB cells. CD36, an important factor for fatty-acid uptake, was not changed under diabetic developmental conditions, neither in EB nor in TB cells (Table 1).

In the EB cells from diabetic rabbits, the protein amount of the phosphorylated ACC was increased compared with that of the normoinsulinemic control rabbits (Figure 3B). The key enzyme for fatty-acid synthesis, FASN, which is not regulated at the mRNA level [35], decreased at the protein level by 20% in the TB (Figure 3C). The expression of SCD1, which catalyzes the rate-limiting step in the formation of monosaturated fatty acids, decreased in both EB and TB cells from diabetic rabbits (Figure 3D).

### 2.4. Stress Marker Expression in Blastocysts from Diabetic Rabbits In Vivo

**Figure 4:**
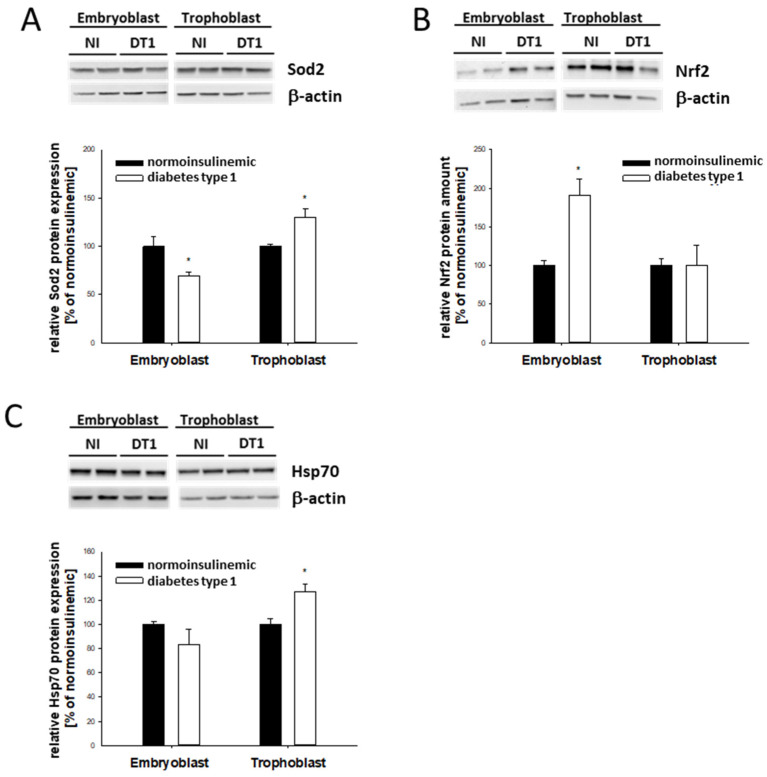
Relative protein abundance of Sod2, Nrf2, and Hsp70 in blastocysts from diabetic rabbits. Protein abundance of Sod2 (**A**), Nrf2 (**B**), and Hsp70 (**C**) was quantified in 6-day-old blastocysts from diabetic [DT1, white bars] and normoinsulinemic [NI, black bars] rabbits. Representative Western blots are shown with two experimentally independent samples. Quantification was performed in at least 3 independent experiments with 8 to 10 blastocysts per sample (N ≥ 3; *n* = 8–10). Relative amounts are shown in bar diagrams after normalization relative to levels of β-actin. Values are expressed as mean ± SEM in % of non-diabetic controls. (*n* ≥ 8; * *p* < 0.05).

Since both PPARα and PPARγ are regulatory factors involved in cellular ER and oxidative stress responses, the markers Sod2, Nrf2, and Hsp70 were analyzed. Sod2 protein was differentially affected by diabetic conditions: it was downregulated in EB and upregulated in TB cells (Figure 4A).

Nrf2, a marker for oxidative stress, increased by almost 100% in EB cells and was unchanged in TB cells (Figure 4B). In contrast, Hsp70 was upregulated by about 20% in the TB cells only (Figure 4C).

### 2.5. Lipid-Dependent Regulation of Intracellular Lipid Accumulation in In Vitro-Cultured Blastocysts

**Figure 5:**
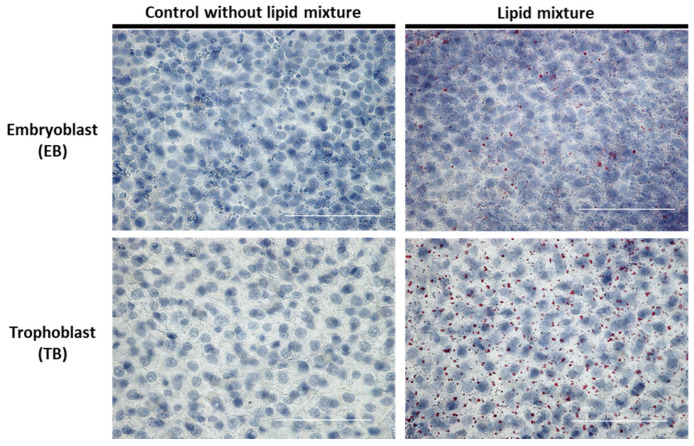
Intracellular lipid accumulation in blastocysts cultured in vitro in media containing a lipid mixture. Six-day-old blastocysts were cultured in vitro with a lipid mixture for 6 h. Lipid droplets were stained red with Oil Red O (red dots). Nuclei were counterstained blue with hematoxylin. Representative images from EB and TB cells are shown (scale bar = 50 µm). Experiments were performed in groups of at least 4 blastocysts in each treatment group and repeated in three independent replicates (N = 3; *n* ≥ 4).

To elucidate whether the changes we observed were directly caused by a high external lipid load, rabbit blastocysts were cultured for 6 h with a commercially available lipid mixture. First, we analyzed whether external lipids alter intracellular lipid accumulation in rabbit blastocysts. EB and TB cells showed a higher abundance of red-stained lipid vesicles compared with the control without lipids (Figure 5).

### 2.6. Lipid-Dependent Expression of Lipogenic Marker Genes in In Vitro-Cultured Blastocysts

**Figure 6:**
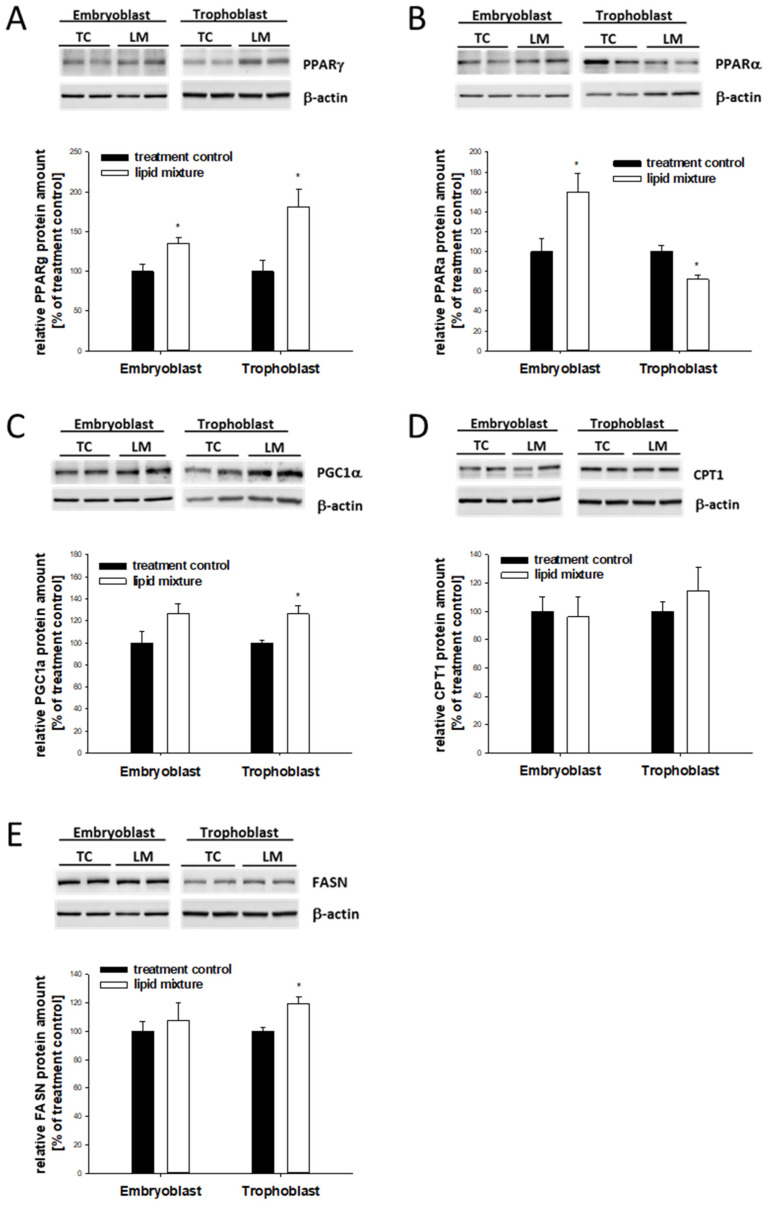
Relative expression of lipogenic genes in rabbit blastocysts cultured in vitro in media containing a lipid mixture. Six-day-old blastocysts were cultured in vitro in groups of 4–5 blastocysts (*n* = 4–5) in media with a lipid mixture (LM) and without (TC) for 6 h. The stimulation experiment was repeated at least three times (N ≥ 3). Protein levels of PPARγ (**A**), PPARα (**B**), PGC1α (**C**), CPT1 (**D**), and FASN (**E**) were quantified using Western blot. Relative amounts are shown as mean ± SEM (* *p* < 0.05). Controls without the lipid mixture were set to 100%.

EB and TB cells responded to the high lipid environment differently. In the EB cells, genes important for fatty-acid uptake, CD36 and FATP4, and binding, FABP4, as well as PPARα and PPARγ, were increased (Table 2 and Figure 6A,B).

This model closely reflected the regulation pattern observed in the in vivo hyperlipidemic environment. In the TB, FABP4 and PGC1α were elevated (Table 2 and Figure 6C). Gene transcription of PPARα, CPT1, and FASN was decreased (Table 2). At the protein level, CPT1 abundance was not altered, while FASN was increased due to the hyperlipidemic in vitro culture conditions (Figure 6D,E).

### 2.7. Lipid-Dependent Expression of Stress Markers in In Vitro-Cultured Blastocysts

**Figure 7:**
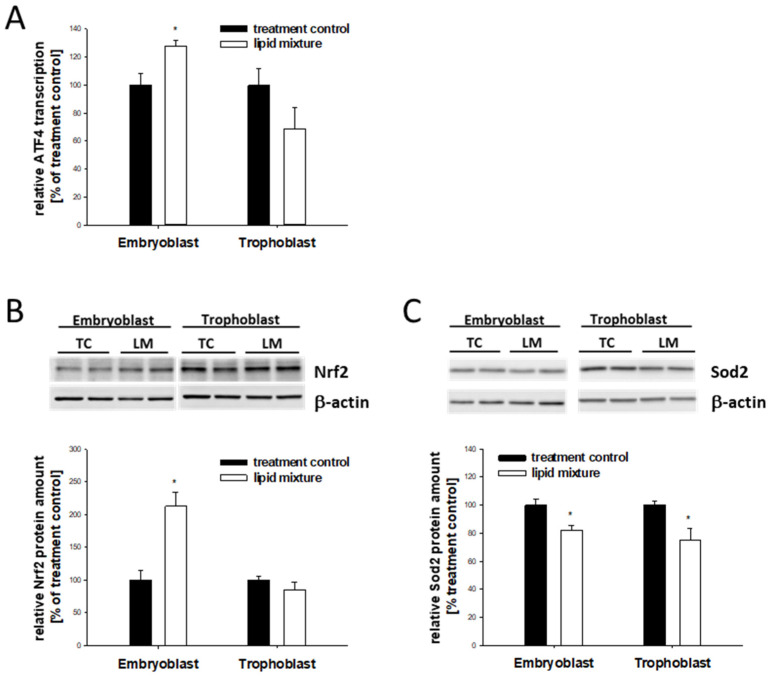
Relative expression of stress markers in rabbit blastocysts cultured in vitro in media containing a lipid mixture. Six-day-old blastocysts were cultured in vitro in groups of 4–5 blastocysts (*n* = 4–5) in media with a lipid mixture (LM) and without (TC) for 6 h. The stimulation experiment was repeated at least three times (N ≥ 3). Gene transcript levels of ATF4 (**A**) and protein levels of Nrf2 (**B**) and SOD2 (**C**) were quantified with RT-PCR (**A**) or Western blot (**B**,**C**). Relative amounts are shown as mean ± SEM (* *p* < 0.05). Controls without the lipid mixture were set to 100%.

Similar to the in vivo results, blastocysts cultured in vitro with the lipid mixture showed an increased protein abundance of ATF4 and Nrf2 and reduced Sod2 in the EB (Figure 7). No changes in ATF4 and Nrf2 were observed in TB cells (Figure 7A,B). In contrast to the regulation pattern observed in vivo, Sod2 was decreased in the TB cells (Figure 7).

## 3. Discussion

Preimplantation embryos are remarkably sensitive to their environment, which influences signaling pathways and gene regulatory networks. Diabetes mellitus impairs maternal and embryonic metabolism, leading to increased intracellular lipid accumulation in day 6 blastocysts, with a more pronounced effect in the EB cells [35]. However, lipid droplets are more than just a ‘storage unit’ for lipids. Recently, Mau and co-workers demonstrated that the accumulation and mobilization of lipid droplets are causal in morphogenesis of the pluripotent epiblast [39]. The excessive amount of lipid droplets in blastocysts from diabetic rabbits can be due either to an increased production of lipids by de novo lipogenesis or by an increased uptake of fatty acids into the cell, leading to an ectopic lipid accumulation. We have previously shown enhanced uptake of fatty acids in blastocysts from diabetic rabbits, as indicated by the increased expression of FATP4 and FABP4 [35]. These genes are expressed in mouse blastocysts [40] and human TB cells [41,42], indicating the importance of these transporters for early embryo development. In the current study, in vitro hyperlipidemic conditions increased the expression of fatty acid transporters and binding proteins predominantly in the EB cells. This observation may explain why lipid accumulation is more increased in EB than in TB under diabetic conditions [35]. Aside from the active uptake of fatty acids by protein-facilitated transfer, passive diffusion through the lipid bilayer is also possible, but this process is not fully understood. Further studies are required to trace fatty-acid movement to determine whether they are taken up passively and if this mechanism is altered in diabetic pregnancy.

The upregulation of fatty-acid transporters and binding proteins correlates with an increased abundance of PPARγ and PPARα in EB cells. PPARs serve as major transcriptional sensors of fatty acids and regulate the expression of FATP4, FABP4, and CD36 [11,12]. FABP4 has a particular importance in lipid metabolism. In addition to transporting fatty acids into the mitochondria for β-oxidation or to lipid droplets for intracellular storage, FABP4 transports fatty acids into the nucleus to act as ligands for the transcription factor PPARγ [43,44]. Blastocysts from diabetic rabbits show a more intense staining of FABP4 in the nuclei compared with healthy control rabbits [35], indicating an enhanced interaction of FABP4 and PPARγ. PPARγ promotes lipid droplet formation and lipid metabolism in the placenta [45]. The observed increased abundance of intracellular lipids, especially in the EB cells of rabbit blastocysts [35], may be a direct result of elevated FABP4 and PPARγ expression.

PPARα regulates fatty-acid uptake mainly through the modulation of CD36 and CPT1 expression, leading to altered β-oxidation [46]. A previous study showed that the overall protein expression of CPT1, the key enzyme in β-oxidation, increased in whole blastocysts in diabetic pregnancy [47]. A different picture emerged when EB and TB cells were analyzed separately. In EB cells from diabetic rabbits, the expression of PPARα and CPT1 was increased, indicating that β-oxidation is likely higher. In TB cells, CPT1 was significantly downregulated. β-oxidation is an essential metabolic pathway for preimplantation embryo development in various species [48,49,50,51]. The inhibition of CPT1 during oocyte maturation and zygote cleavage impairs subsequent blastocyst development [49]. In the current study, a diabetic hyperlipidemic environment supported β-oxidation in EB cells, which may have long-lasting effects on further embryo development.

De novo lipogenesis is essential for proper embryo development. For example, deletion of FASN results in embryonic lethality [52]. Together with ACC, FASN regulates the lipogenic flux from malonyl-CoA into palmitate. In blastocysts from diabetic rabbits, gene activation of ACC and a lower abundance of FASN and SCD1 were observed in EB and TB cells from diabetic rabbits, demonstrating a possible reduction in endogenous palmitate production and conversion of palmitate and stearate into monounsaturated fatty acids [53]. Insulin can regulate SCD1 and FASN via SREBP1c in human cells and sheep [54,55]. The lack of insulin is directly connected to the observed lower abundance of SCD1 and FASN in embryos from diabetic mothers.

Ectopic lipid accumulation is increased in response to a high lipid environment, leading to damage to cell organelles, particularly in the ER and mitochondria [56,57,58]. In mitochondria, the elevation of β-oxidation produces higher reactive oxygen species, impairing normal mitochondrial function and leading to DNA damage [59,60,61]. In addition to direct effects on the redox state, PPARγ and PGC1α also regulate the expression of anti-oxidative enzymes such as Sod2 and Nrf2. Sod2 gene expression is modulated by PPARγ via the transcription factor cAMP-responsive element binding protein (CREB) [18,62]. In rabbit blastocysts, CREB is regulated in a cell lineage-specific manner [31]. In the EB cells, CREB activity was increased due to maternal diabetes. In the TB cells, CREB was inactive. The oxidative stress marker Nrf2 is modulated by PPARγ via PGC1 [21,22,63]. In EB cells, the expression of PPARγ and PGC1α were positively correlated with Nrf2 protein expression. Oxidative stress can diminish cell membrane integrity, organelle function, and the regulation of gene expression, thereby contributing to cell death [64]. Therefore, the induction of Nrf2 may contribute to increased apoptosis, higher embryo loss, and developmental delay in diabetic pregnancies which specifically affect the EB cells [65,66].

ER stress has also been reported in embryos from diabetic mice [27,28,29]. Impaired function of the ER affects protein secretion and the induction of autophagy [64,67,68]. Autophagy activity was altered in blastocysts from diabetic rabbits, as shown by a reduced abundance of p62, a marker for autophagic activity and lysosomal vesicles [34]. ATF4 is another biomarker for ER stress [30]. ATF4 is expressed in the rabbit preimplantation embryo, and maternal diabetes mellitus led to increased transcription of ATF4 in the EB cells [31]. In the current study, we could show via in vitro experiments that the increase in ATF4 transcription could be related to ectopic lipid accumulation. In a mouse model, depletion of ATF4 resulted in reduced expression of PPARγ, SREBP1c, and FASN [69], indicating that changes in ATF4 abundance could directly affect intracellular lipid metabolism. These results imply that ER stress and its triggered adaptive response mechanism can affect lipid metabolism.

Blastocyst metabolism regulates more than the steady energy supply from ATP production. It is becoming increasingly evident that exogenous metabolites and cofactors are important regulators of embryo fate, putting environmental interactions at the forefront. Therefore, alterations of the surrounding milieu of the preimplantation embryo can have profound implications for an organism’s health and predisposition to diseases later in life. We have shown that maternal diabetes mellitus affects embryonic lipid metabolism in a cell lineage-specific manner, leading to an altered stress response in EB and TB cells only a few hours after gastrulation has started. This effect can be explained in part by diabetes-associated hyperlipidemia. In summary, preimplantation embryo development is a vulnerable period in an individual’s life and can program adult disease susceptibility. Therefore, changes in embryonic metabolism and stress response may have severe consequences for future health trajectories.

## 4. Materials and Methods

### 4.1. Alloxan Treatment

Experimental insulin-dependent diabetes was induced in mature 18–20-week-old female non-pregnant rabbits by alloxan treatment (Sigma-Aldrich, Darmstadt, Germany), as described previously [65]. Rabbits were held in a diabetic condition with permanent blood glucose concentrations > 14 mmol/L by regular insulin supplementation three times per day (Huminsulin^®^ Basal, Lilly Deutschland GmbH, Bad Homburg, Germany), starting at the second day after alloxan treatment. Blood glucose level was monitored as described previously [32].

### 4.2. Embryo Recovery and In Vitro Culture

Mating and embryo recovery were performed as described [31]. At day 6 p.c., rabbits were euthanized with a lethal dose of sodium pentobarbital. Embryos were flushed from the uteri, washed three times with basal synthetic medium II (serum- and growth-factor-free) [70], characterized morphologically using a stereomicroscope, and classified for gastrulation stages [71]. Gastrulation stages 1 (gastrulation stage with anterior marginal crest) and 2 (gastrulation stage with posterior gastrulation extension) were used for in vivo analysis and in vitro culture.

In vitro culture of day 6 blastocysts was performed in groups of 4 to 5, if embryos were used for RNA analyses or 9 to 10 for protein analyses. In vitro culture was performed at 37 °C in a water-saturated atmosphere of 5% O_2_, 5% CO_2_, and 90% N_2_, for 6 h with a commercial lipid mixture (chemically defined lipid mixture #11905, Thermo Fisher, Dreieich, Germany) diluted 1:100 as recommended in standard culture medium. The lipid mixture contained fatty acids, cholesterol, and phospholipids and has been used in other studies to create hyperlipidemic developmental conditions [72,73]. Control embryos were cultured without the lipid mixture in standard culture medium.

Embryos were washed three times in cold phosphate-buffered saline (PBS) containing 0.05% polyvinyl alcohol (PVA). Embryonic coverings were mechanically removed and blastocysts were microdissected to harvest EB and TB cells under a stereomicroscope. RNA analyses were performed using single embryos stored in PBS at −80 °C until RNA isolation for RT-PCR. For protein analyses, EB and TB cells were pooled in groups of 8–10 for one protein sample. Samples were stored at −80 °C until use in PBS buffer for RNA isolation and radioimmunoprecipitation assay (RIPA) buffer for protein isolation.

### 4.3. Staining of Rabbit Blastocysts

Blastocysts have been describe previously [35]. In brief, fixed blastocysts were used immediately after recovery for Oil Red O (Sigma-Aldrich) staining. Embryonic tissues were stained for 2 h, washed in 0.05% (wt/vol) PVA/PBS and embedded on Superfrost^TM^ slides (Menzel Gläser, Braunschweig, Germany) using 4.8 g of MOWIOL^®^ reagent (Merck, Darmstadt, Germany) dissolved in 12.0 g of glycerol (Merck, Darmstadt, Germany). Embryonic disks were examined using light microscopy (BZ 8000, Keyence, Itasca, IL, USA).

### 4.4. RNA Isolation and cDNA Synthesis

Dynabeads^TM^ Oligo(dT) 25 (Invitrogen, Darmstadt, Germany) were used to isolate mRNA from single EB and TB cell samples. All isolated mRNA was further used for cDNA synthesis. All procedures were carried out according to the manufacturer’s instructions with previously described modifications by [74]. Whole mRNA samples were transcribed into cDNA using RevertAid^TM^ H Minus Reverse Transcriptase (200 U/µL) (Thermo Fisher Scientific, Dreieich, Germany) in a thermocycler (Biometra, Göttingen, Germany) as follows: 10 min at 25 °C, 1 h at 42 °C, and 10 min at 70 °C. Sterile water was added to the samples to a final volume of 100 µL.

### 4.5. Polymerase Chain Reaction (RT-PCR)

RT-PCR amplification was conducted with 0.5 µL cDNA from single blastocysts in 25 µL volume containing 200 µM of each dNTP, 2.5 U Taq polymerase, and specific oligonucleotides for PPARα, PPARγ, FASN, CPT1, FABP4, FATP4, CD36, and GAPDH (primers listed in Supplemental Table S1). Amplification was performed for 40 cycles (94 °C 45 s, 60 °C 45 s, 72 °C 60 s). PCR products were separated by electrophoresis on 2% agarose gels and stained with ethidium bromide.

### 4.6. Measurement of mRNA Levels Using Quantitative PCR (qPCR)

Amplification- and quantification-specific forward and reverse primers were designed based on rabbit gene sequences using the Primer-BLAST online tool (NIH, Bethesda, MD, USA; Supplemental Table S1). PCR products were sequenced and verified for specificity as described previously [75]. Glyceraldehyde-3-phosphate dehydrogenase (GAPDH) gene expression, which is unaffected by the treatment [76], was quantified as the endogenous control for EB and TB samples. qPCR analyses were performed in duplicate using a Quant Studio 3^TM^ Real Time System (Thermo Fisher, Dreieich, Germany) as previously described [77]. In brief, qPCR reactions were performed using 3 μL of cDNA and 17 μL master mix (PowerTrack™ SYBR Green Master Mix, Thermo Fisher, Dreieich, Germany) and with a ‘no template control’ for each primer set (Supplemental Table S1). Results were calculated as abundances of target RNA molecules per GAPDH RNA molecules and expressed as relative abundances in percent- or fold-change of control samples. Target gene expression is described relative to the mean of the normoinsulinemic group.

### 4.7. Protein Sample Preparation

Protein isolation of pooled blastocysts was performed as described by Pendzialek et al. [78]. In brief, embryonic tissues were homogenized in ice-cold RIPA buffer containing protease and phosphatase inhibitors (Roche, Basel, Switzerland). EB cells were homogenized in 5 μL and TB cells in 20 μL of RIPA buffer per embryo. Protein concentration was determined using the Pierce™ 660 nm Protein Assay (Thermo Fisher Scientific, Dreieich, Germany) according to the manufacturer’s instructions.

### 4.8. Immunoblotting

For Western blot analysis, 25 μL of sample containing 20 µg of EB cells or 45 µg of TB cells was subjected to sodium dodecyl sulfate-polyacrylamide gel electrophoresis (SDS-PAGE) on 10–12% gradient gels and electrotransferred to nitrocellulose membranes. Immunoblotting has been described in [47]. Supplemental Table S2 describes antibody information. Immunoreactive signals were visualized via enhanced chemiluminescence detection and quantified using a ChemiDoc™ Touch System and Image Lab 5.2.1 software (Bio-Rad, Hercules, CA, USA). Relative protein abundance was calculated as the ratio of the band intensity of the target protein to the band intensity of β-actin in the same blot to correct for differences in protein loading.

### 4.9. Statistics

Statistical analyses were performed with SigmaPlot (v. 12.0; Systat Software Inc., San Jose, CA, USA). The level of significance between groups was calculated with a two-tailed Student’s *t*-test after testing for outliers and proving normal distribution. If normal distribution failed, the Mann–Whitney U test was used. Results are shown as mean value ± standard error of the mean (mean ± SEM). Multiple comparisons were made with factorial variance analysis (ANOVA) adjusted according to Bonferroni correction. Results were considered statistically significant if *p* < 0.05. (N) represents the number of individual and independent experiments from which the embryos were covered and (*n*) the number of samples used per measurement per group. All experiments were repeated at least three times.

## Figures and Tables

**Table 1:** Relative mRNA abundance of lipogenic target genes in embryoblast (EB) and trophoblast (TB) cells from normoinsulinemic and diabetic rabbits.

	Relative mRNA Amount (in % of Normoinsulinaemic EB)			
	Embryoblast		Trophoblast	
Gene	Normoinsulinemic	Diabetic	Normoinsulinemic	Diabetic
PPARα	100.0 ± 16.9	116.0 ± 11.8	44.4 ± 8.6 **^b^**	29.1 ± 6.3 **^b^**
PPARγ	100.0 ± 15.1	177.9 ± 35.4 **^a^**	753.1 ± 58.9 **^b^**	528.2 ± 135.8 **^b^**
CPT1	100.0 ± 16.2	125.1 ± 35.4	222.4 ± 58.1	90.8 ± 15.9
CD36	100.0 ± 24.3	60.3 ± 11.3	286.6 ± 29.1 **^b^**	295.4 ± 41.6 **^b^**

Results are shown as [mean ± SEM] in percentage of the amount in the embryoblast gene expression from normoinsulinemic rabbits (N = 3; *n* ≥ 10). ^a^—significantly different between normoinsulinemic and diabetic rabbits (*p* < 0.05). ^b^—significantly different between EB and TB cells (*p* < 0.05).

**Table 2:** Relative mRNA abundance of lipogenic target genes in embryoblast (EB) and trophoblast (TB) cells of 6-day-old rabbit blastocysts after 6 h in vitro exposure with a lipid mixture.

	Relative mRNA Amount [5 of Nontreated Control]			
Gene	Embryoblast		Trophoblast	
mRNA	Treatment Control	Lipid Mixture	Treatment Control	Lipid Mixture
PPARα	100.0 ± 7.8	112.1 ± 11.0	100.0 ± 10.3	70.6 ± 6.6 *
PPARγ	100.0 ± 8.6	169.9 ± 20.4 *	100.0 ± 10.5	101.3 ± 11.7
CPT1	100.0 ± 9.9	136.9 ± 19.7	100.0 ± 13.8	59.1 ± 6.6 *
FASN	100.0 ± 10.9	167.9 ± 49.2	100.0 ± 12.4	49.7 ± 6.4 *
FABP4	100.0 ± 7.1	170.1 ± 23.4 *	100.0 ± 7.8	200.3 ± 24.7 *
FATP4	100.0 ± 11.5	141.0 ± 11.9 *	100.0 ± 13.8	85.2 ± 9.5
CD36	100.0 ± 16.6	238.5 ± 43.9 *	100.0 ± 9.3	104.4 ± 11.2

Results are shown as percentages of the amount in the EB or TB cells in blastocysts without the lipid mixture (control) with mean ± SEM; N = 3; *n* ≥ 10. * abundance significantly different between control and lipid mixture groups (*p* < 0.05).

## Data Availability

The data underlying this article will be shared on reasonable request to the corresponding author.

## Supplementary Materials

The following supporting information can be downloaded at https://www.mdpi.com/article/10.3390/ijms241411776/s1.
