# Supplementary material for: Ectopic Lipid Accumulation Correlates with Cellular Stress in Rabbit Blastocysts from Diabetic Mothers
Source: Int J Mol Sci. 2023 Jul 21;24(14):11776. doi: 10.3390/ijms241411776 (PMC10380447; doi:10.3390/ijms241411776)
Supplement: Supplementary file 1 [file ijms-24-11776-s001.zip › ijms-2491888-supplementary.pdf]

Table S1. **Oligonucleotides used for quantitative real time PCR.**

Name	Primer sequence 5´- 3´	Product [bp]	Acc. No.
**rabbit ATF4**	fw: GCGAGAAGCTGGAGAAGAAG<br>rev: TCCAGCAGGTCCTTGAGGTA	193	NM_001675
**rabbit CD36**	fwd:GGGTGAAAACAGGCACAGAT<br>rev: TGTGTCATTCTCGGTTCCAA	243	AF412572
**rabbit CPT1B**	fwd: GGACAGCTACGCCAAGTCTC<br>rev: AGTGTCCGTCCTCCGTGTAG	203	XM_002723942
**rabbit FASN**	fwd: AGGAACTCCCCTCATCTCCC<br>rev: TTGCCGTTCTCTGACACCTC	378	NM_004104.4
**rabbit FABP4**	fwd: TCAGTGTGAATGGGGATGTG<br>rev: TGCATTCCACCACCAGTTTA	228	XM_002710655
**rabbit FATP4**	fwd: CACCTGTGACTTGGAGCACTT<br>rev: GCAGCTCTGTCTTAAGGAGCTT	131	XM_002722970.1
**rabbit GAPDH**	fwd: GCCGCTTCTTCTCGTGCAG<br>rev: ATGGATCATTGATGGCGACAAAT	144	L23961
**rabbit PPARα**	fwd: TTGTGGCTGCTATCATCTGC<br>rev: GAGTTTGGGAAGAGGAAGG	146	XM_002723354.3
**rabbit PPARγ**	fwd: AGATCATCTACACCATGCTGGCCT<br>rev: TGTCCTCGATGGGCTTCACATTCA	252	NM_001082148.1

Gene names, forward (fwd) and reversed (rev) primer sequences, PCR product length (in base pair (bp)) and accession numbers (Acc.No.) for the sequence of origin in GenBank® are listed in alphabetical order. Annealing temperature (Tm) for all primer combinations was 60 C°.

Table S2. **List of antibodies for Western Blot.**

Protein target	Antibody	Manufacturer, # catalog, antibody type	Dilution used
**pACC**	Phospho-Acetyl-CoA Carboxylase (Ser79)	Cell signaling, #3661, RM	1:250 (EB), 1:250 (TB)
**CPT1B**	CPTI antibody (E-7)	Santa cruz, #sc-393070, MM	1:100 (EB), 1:100 (TB)
**HSP70**	HSP70 antibody (3A3)	Santa cruz, #sc-32239, MM	1:200 (EB), 1:500 (TB)
**FASN**	Fatty Acid Synthase antibody (A-5)	Santa cruz, #sc-55580, MM	1:100 (EB), 1:200 (TB)
**Nrf2**	Nrf2 antibody (A-10)	Santa cruz, #sc-365949, MM	1:125 (EB), 1:250 (TB)
**PGC1α**	PGC1 alpha antibody	Abcam, #ab77210, MM	1:125 (EB), 1:250 (TB)
**PPARγ**	PPARγ antibody (E-8)	Santa cruz, #sc-7273, MM	1:100 (EB), 1:100 (TB)
**PPARα**	PPARα antibody (H-2)	Santa cruz, #sc-398394, MM	1:100 (EB, TB)
**SCD1**	SCD1 Antibody (D-5)	Santa cruz, # sc-515875, MM	1:125 (EB), 1:250 (TB)
**SOD2**	SOD-2 antibody (B-1)	Santa cruz, B-1, #sc-133254, MM	1:200 (EB), 1:500 (TB)
**β-Actin**	Monoclonal anti-β-actin antibody clone AC-15	Sigma, #A5441, MM	1:20000
**Anti-rabbit**	Polyclonal goat anti-rabbit Immunoglobulin/HRP	Dako, #P0448	1:10000
**Anti-mouse**	Goat anti-mouse IgG (H+L)-HRPO	Dianova, #115-036-003	1:50000

BSA: bovines serum albumin; DP: donkey polyclonal antibody; IgG P: IgG polyclonal antibody; MM: mouse monoclonal antibody; MP: mouse polyclonal; RM: rabbit monoclonal antibody;

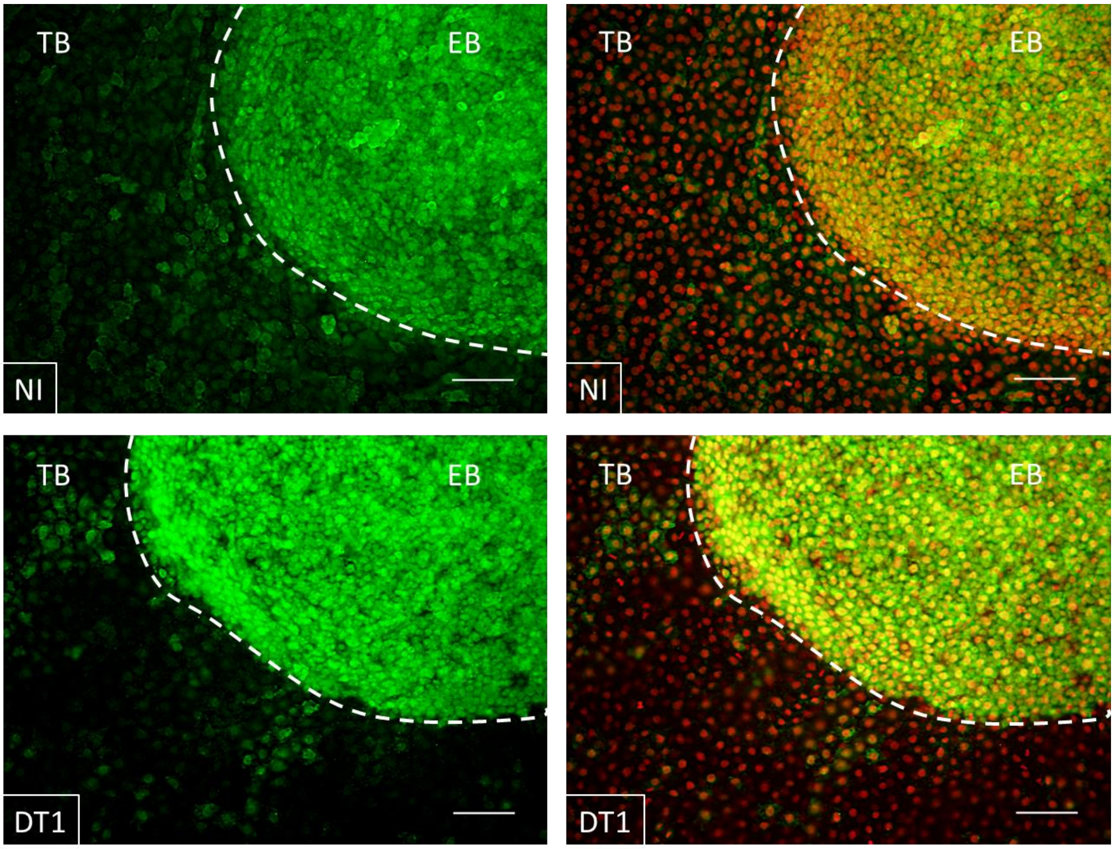

Figure S1. **Whole mount staining of PPARα in day 6 blastocysts.**

Immunofluorescent detection of PPARα (green colour) was performed in day 6 blastocysts (st.2) from normoinsulinaemic (NI) and diabetic (DT1) rabbits.
Blastocysts were washed twice in ice-cold PBS and fixed in 4% (wt/vol) paraformaldehyde at 4°C over night. Preparation and immunhistochemical protocol were performed as described (Schindler et al. 2013, Schindler et al. 2013). Antibody for PPARa (#sc-393070, santa cruz, Germany) were diluted 1:100 in 3% (wt/vol) bovine serum albumin (BSA)/PBS. The secondary antibody conjugated with Alexa fluor 488 (Alexa fluor® 488 Goat Anti Mouse, Invitrogen, Germany) was diluted 1:300 in PBS. The nuclei were counterstained with 7-AAD (1:100 diluted in PBS, Molecular Probes, Eugene, USA). The secondary antibody and 7-AAD was incubated for 1 h. All steps were performed within the same experiment, examined microscopically during the same session, using identical microscope and camera settings (BZ 8100, Keyence).
PPARα was localized in the nuclei of embryoblast (EB) cells with a brighter staining in DT1 rabbits. Trophoblast (TB) was only weakly stained for PPARα in both NI and DT1 rabbits (scale bar=100µm). Nuclei were counterstained with 7-AAD (red). Signal overlay of PPARα and 7-AAD results in yellow colour and demonstrate the nuclear localisation of PPARa. In EB in almost all cells PPARa was localised in the nucleus (n=8).
